# Analysis of the Structure of Surgical Activity for a Suturing and Knot-Tying Task

**DOI:** 10.1371/journal.pone.0149174

**Published:** 2016-03-07

**Authors:** S. Swaroop Vedula, Anand O. Malpani, Lingling Tao, George Chen, Yixin Gao, Piyush Poddar, Narges Ahmidi, Christopher Paxton, Rene Vidal, Sanjeev Khudanpur, Gregory D. Hager, Chi Chiung Grace Chen

**Affiliations:** 1 Department of Computer Science, The Johns Hopkins University, Baltimore, Maryland, United States of America; 2 Department of Electrical and Computer Engineering, The Johns Hopkins University, Baltimore, Maryland, United States of America; 3 Department of Biomedical Engineering, The Johns Hopkins University, Baltimore, Maryland, United States of America; 4 Department of Gynecology and Obstetrics, Johns Hopkins University School of Medicine, Baltimore, Maryland, United States of America; The Chinese University of Hong Kong, HONG KONG

## Abstract

**Background:**

Surgical tasks are performed in a sequence of steps, and technical skill evaluation includes assessing task flow efficiency. Our objective was to describe differences in task flow for expert and novice surgeons for a basic surgical task.

**Methods:**

We used a hierarchical semantic vocabulary to decompose and annotate maneuvers and gestures for 135 instances of a surgeon’s knot performed by 18 surgeons. We compared counts of maneuvers and gestures, and analyzed task flow by skill level.

**Results:**

Experts used fewer gestures to perform the task (26.29; 95% CI = 25.21 to 27.38 for experts vs. 31.30; 95% CI = 29.05 to 33.55 for novices) and made fewer errors in gestures than novices (1.00; 95% CI = 0.61 to 1.39 vs. 2.84; 95% CI = 2.3 to 3.37). Transitions among maneuvers, and among gestures within each maneuver for expert trials were more predictable than novice trials.

**Conclusions:**

Activity segments and state flow transitions within a basic surgical task differ by surgical skill level, and can be used to provide targeted feedback to surgical trainees.

## Introduction

Graduate training programs and professional re-certification ensure that competent individuals deliver surgical care to patients. Surgical competence is determined by many factors, including cognitive ability, personality traits, and psychomotor (technical) skills, the last of which are acquired through deliberate practice [1,2]. Inferior surgical (technical) skills are associated with higher incidence of post-operative complications, including re-operation, re-admission, and death [3,4]. Increasing concerns for safety, effectiveness, and quality of surgical care amidst new limits on resident training hours and rising time-demands on surgical educators are driving the need for new tools for surgical education and surgical skills assessment [5,6]. Furthermore, objective measures for surgical skill and competency are important as medicine becomes increasingly driven by accountability and transparency.

Decomposition of structured surgical tasks into their component steps or activity segments may be used to provide targeted feedback to trainees, i.e., where in the task the surgeon should improve upon their skill [7–9]. Decomposition of surgical activity, for example applying cognitive task analysis, is often used for teaching surgical technical skills [10–13]. Surgical task flow (the sequence of steps in which the task is performed) represents an important aspect of surgical technical skill and is typically used to assess efficiency, autonomy and knowledge of task [14,15]. A previous study examined differences between a single expert and four novice surgeons in the duration of activity segments for simple surgical tasks in a porcine model [16]. Our objective was to compare activity segments and task flow between expert and novice surgeons while they performed a surgeon’s knot on a bench-top model using the *da Vinci* Surgical System^®^ (dVSS; Intuitive Surgical, Inc., Sunnyvale, CA).

## Materials and Methods

### Dataset

We captured video and tool motion data from the dVSS as 18 surgeons performed repetitions of a study task, composed of a single suture throw and a 2–1 surgeon’s knot on an inanimate bench-top model (a two loop knot followed by a one loop knot). Details about the collection and curation of the dataset are described elsewhere [17,18]. Briefly, four expert surgeons (attending surgeons with robotic surgery practices) and 14 trainee surgeons (who had no or limited experience with robotic surgery) performed the study task in multiple sessions over several weeks. The surgeons performed three repetitions (i.e., “trials”) of the study task in each session, which we analyzed as three separate data points. We captured data from the dVSS, including stereo video (at 30 frames per second), kinematic data describing motion of arms on both the surgeon’s console and patient side of the robot (at 50 Hertz), and system/user events such as the use of clutch and camera [17,18].

### Generation of vocabulary for activity segments

**Fig 1:**
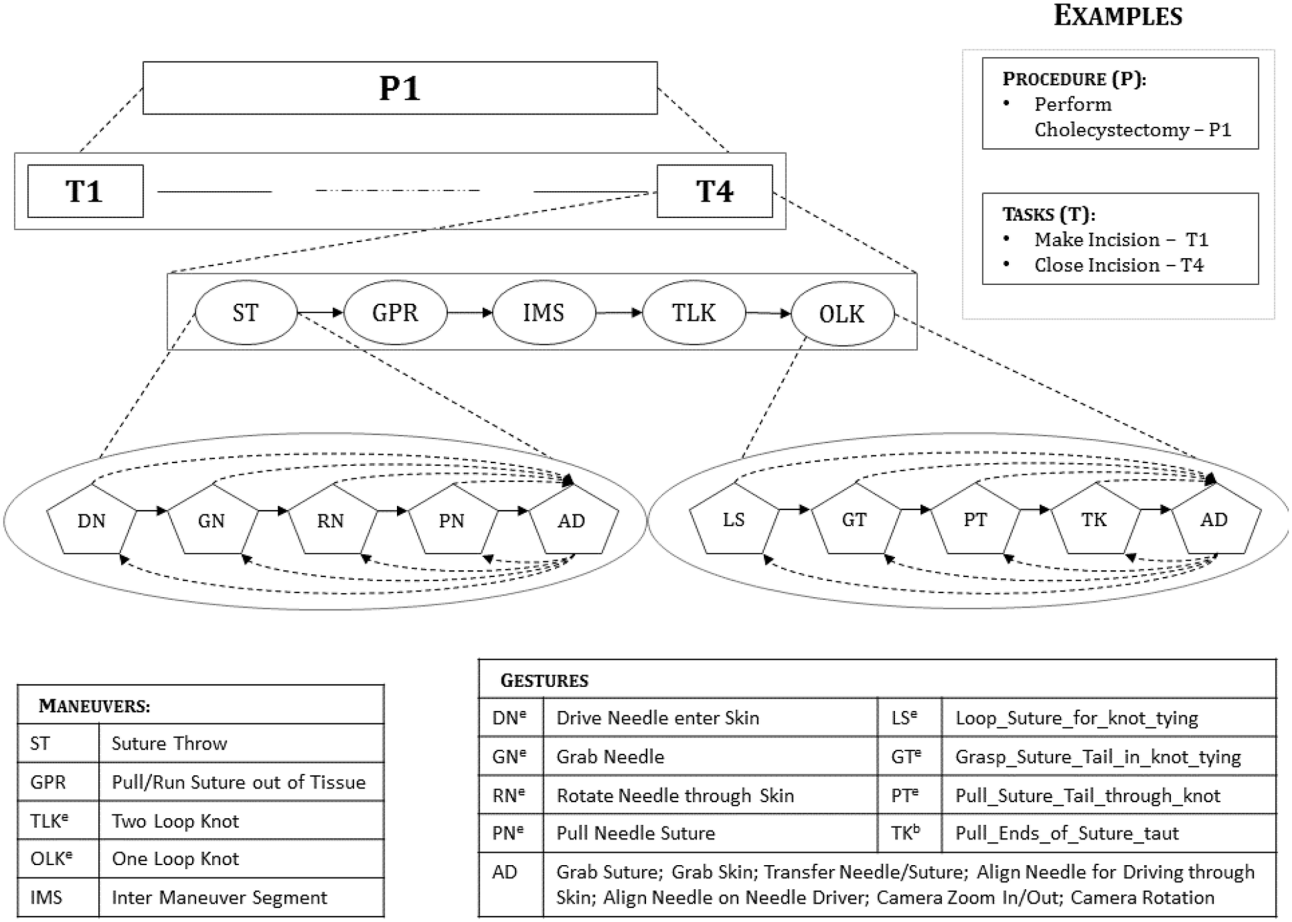
Hierarchical semantic decomposition of surgical activity. ^e^ denotes that the segment can be performed using either of the robotic arms, ^b^ denotes that the segment is performed using both the robotic arms.

We used a vocabulary based on a hierarchical segmentation of the study task for annotating segments of surgical activity as shown in Fig 1. We divided the task into maneuvers, which we defined as circumscribed activity segments that accomplish specific landmarks (e.g., tying a knot) in completing the surgical task. We further decomposed maneuvers into gestures. We defined gestures as atomic segments of activity performed to complete a meaningful portion of the task (e.g., looping suture around needle driver in a knot tying maneuver). The hierarchical structure of surgical activity indicates that maneuvers may be shared across tasks and gestures may be shared across maneuvers. Hereafter, we use “activity segments” to generically refer to both maneuvers and gestures.

Our goal was to develop a vocabulary of non-overlapping semantic gesture labels such that each label defined a specific activity, the tool performing the activity, and the object with which the tool interacts while performing the activity. We generated the gesture vocabulary using transcriptions of an expert surgeon’s narration as she watched videos of the study task being performed. We used the transcriptions to identify potential labels that describe unit segments of surgical activity. We refined the potential labels through discussions with the surgeon to generate an initial gesture vocabulary. We further revised the initial gesture vocabulary to add four more gestures identified while annotating videos of the study task.

We generated the maneuver vocabulary using a process similar to that for gestures, through consultations with an expert surgeon. We annotated each trial for seven maneuvers—suture throw (ST), grasp-pull-run maneuver to run suture through tissue (GPR), two-loop knot (TLK) using each robotic arm, one-loop knot (OLK) using each robotic arm, and inter-maneuver segment (IMS). IMS includes activity that helps the surgeon to optimally position the tools for the next maneuver. For our analyses, we collapsed the maneuvers into five labels—ST, GPR, TLK, OLK, and IMS.

### Gesture and maneuver annotation

One individual annotated each trial in our dataset using the initial gesture vocabulary using a custom-designed software. Subsequently, another individual verified and revised annotations by the first individual using the final gesture vocabulary (hereafter referred to as “verified annotations”). For a subset of trials in our dataset, two individuals independently annotated videos with gesture labels using the initial gesture vocabulary (hereafter referred to as “independent annotations”). Gesture annotations included the start and end of the gesture, and whether the gesture was performed with error. Two investigators independently annotated maneuvers using the gesture label transcripts. Maneuver annotations included the start of the first gesture and end of the last gesture comprising the maneuver, and whether the maneuver was complete.

### Surgical skill annotation

One expert surgeon reviewed video recordings for each session (three trials) performed by every operator using a modified global rating score (GRS) using the Objective Structured Assessment of Technical Skills (OSATS) approach [15,17]. We used the respect for tissue, time and motion, instrument handling, knowledge of instruments, flow of operation, and knowledge of specific procedure elements from the original approach, and omitted the “use of assistants” item because it is not applicable to our study task.

We assigned trials to skill categories using two methods—self-reported experience and an expert-assigned GRS. Using self-reported experience, we defined attending surgeons with robotic surgery practices as “experts” and trainee surgeons as “novices” in robotic surgical skills. We considered trials with GRS less than 14 as having been done by a “novice”, and with GRS greater than 22 as having been done by an “expert”, and the rest as having been done by “intermediate” level surgeons (GRS definition #1).

There are no accepted cut-offs for GRS using the OSATS approach to distinguish novice and experts. Therefore, in sensitivity analyses, we used two alternative GRS-based skill definitions—first, considering trials with a score of three or more on at most two items in GRS as “novice,” trials with a score of more than three on at least four items in GRS and a score of at least three on the remaining two items as “expert,” and the rest as “intermediate” (GRS definition #2); and the second, considering trials with a total GRS at most three on all items as “novice,” trials with a GRS more than 3 on all items as “expert,” and the rest as “intermediate” (GRS definition #3). Our findings and conclusions were similar with all three definitions for GRS-based skill categories, and so we report our findings using GRS definition #1.

### Analysis of inter-annotator reliability

We assessed inter-annotator reliability by measuring agreement on both the sequence of labels assigned to each trial and the gesture annotation for each frame in the trial. We computed the Levenshtein’s distance (LD) [19] to measure inter-annotator agreement on the sequence of labels assigned to each trial. LD is a commonly used metric to compare two strings. It quantifies the number of edits (i.e., deletions, insertions, or substitutions) required to convert one string into another. For example, the LD comparing “robots” and “robust” will be three—one for substituting “o” with “u,” one for inserting “s” after the substituted letter “u,” and one for deleting the “s” at the end of the word “robots”. To measure inter-annotator agreement on the gesture label assigned to each frame in a trial, we computed a Cohen’s kappa [20]. Because a given frame may be labeled using more than one gesture label, we computed the kappa separately for each category of gestures including those specific to each robot arm, gestures that require both the robot arms, and for gestures describing camera movements.

### Analysis of task flow by level of surgical skill

For our descriptive analyses, we computed segment counts and their 95% bootstrap intervals (95% BI; using 1000 bootstrap samples) for the overall trials by surgical skill level using both experience-based and GRS-based skill definitions, for total numbers of maneuvers, incomplete maneuvers, IMS, gestures, error gestures, for total gestures within maneuvers, and for specific gestures within maneuvers (highlighted in red in Fig 1) including driving needle (DN), grasping needle (GN), rotating needle out of tissue (RN), pulling needle (PN), adjustment gestures (AD), looping suture around needle-driver (LS), grasping suture tail through loop in a knot (GT), pulling suture tail through loop in a knot (PT), and pulling both suture ends to tighten knot (TK). We considered differences between groups to be statistically significant when the estimate in one group was not within the 95% BI for the other group.

We compared transitions among maneuvers and among gestures within ST, TLK, and OLK maneuvers between trials by expert and novice surgeons (experience-based skill definition). For this comparison, we first computed the transition probability between activity segments using trials within each skill level. Elements in the transition probability matrix are probabilities of one activity segment following another. We used the transition probability matrices to generate state-flow plots for each skill level. We visually compared the plots to describe differences in task flow by skill level. We then compared the probability distributions of transitions emanating from each maneuver between experts and novices by computing the Hellinger distance between the distributions [21]. If expert and novice surgeons perform the task using the same sequence of steps then the Hellinger distance between the transition probability distributions will be zero. We computed a 95% BI for the Hellinger distance estimated on the original dataset using 1000 bootstrap samples.

Finally, we determined whether experts performed the task and certain maneuvers (ST, TLK, OLK) using a more predictable sequence of activity segments compared with novice surgeons (experience-based skill definition). For this comparison, we computed the difference in conditional entropy for the probability distribution of transitions among activity segments for trials by expert and novice surgeons. If the transitions out of a particular activity segment are completely predictable then entropy equals zero and if all transitions out of the activity segment are equally probable (uniform probability distribution) then the value of entropy will be at its maximum. Thus, the conditional entropy of the segment transition probability matrix of trials for a certain skill level represents the predictability in the sequence of segments for trials in that skill level. We computed a 95% BI for the difference in conditional entropy for trials by expert and novice surgeons using 1000 bootstrap samples. We used MATLAB^®^ (The MathWorks, Inc., Natick, MA) for our analyses and Graphviz [22] for state flow plots.

## Results

Our vocabulary for describing activity segments in our study task included seven maneuvers and 30 gestures (Fig 1). Maneuver sequences obtained from two separate annotators differed by an average LD of 0.30 labels per 10 maneuver labels in a trial (95% CI = 0.16 to 0.44). Gesture sequences obtained from two separate annotators differed by an average LD of 1.34 labels per 10 gesture labels in a trial (95% CI = 1.02 to 1.64). We observed high agreement (kappa) for maneuvers and moderate to high inter-annotator agreement for gestures (Table A in S1 File).

### Analysis of maneuver counts by surgical skill

**Fig 2:**
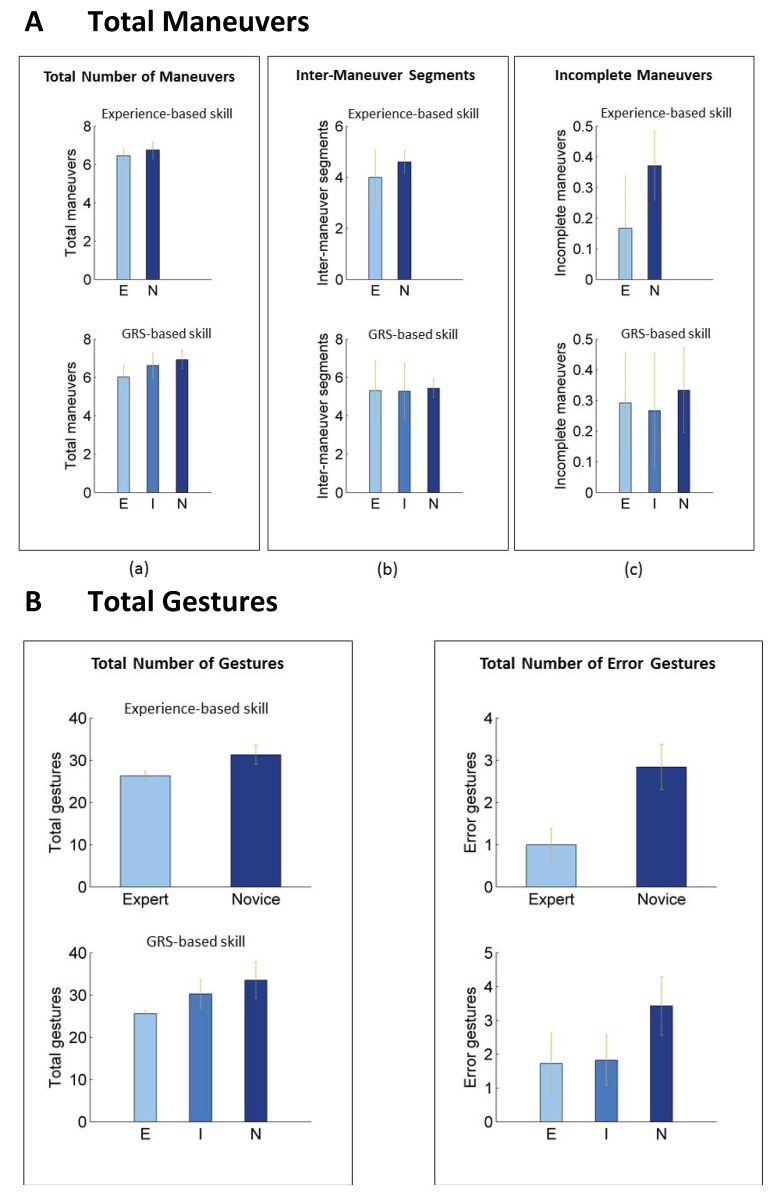
Maneuver and gesture counts for overall task by skill level. GRS = global rating score; E = expert; I = intermediate; N = novice. The error bars illustrate 95% bootstrap intervals. We observed statistically significant differences in the total count for maneuvers and the count for incomplete maneuvers between expert and novice trials by GRS-based definition but not using experience based-definition for surgical skill. We observed statistically significant differences in the total count for gestures and error gestures between expert and novice trials per both experience- and GRS-based definitions for surgical skill.

Trials by expert surgeons (experience-based skill definition) were performed using fewer maneuvers over the entire task than trials by novice surgeons; the difference between the groups was not statistically significant (Fig 2, Table B in S1 File). Trials graded as expert by GRS were performed using fewer maneuvers than trials graded as intermediate or novice; only the difference between expert and novice trials was statistically significant.

Trials by expert surgeons (experience-based skill definition) were also performed with fewer incomplete maneuvers than trials by novice surgeons; the difference between the groups was statistically significant. Differences in the numbers of incomplete maneuvers were not statistically significant across trials in different GRS categories.

### Analysis of gesture counts by surgical skill

**Fig 3:**
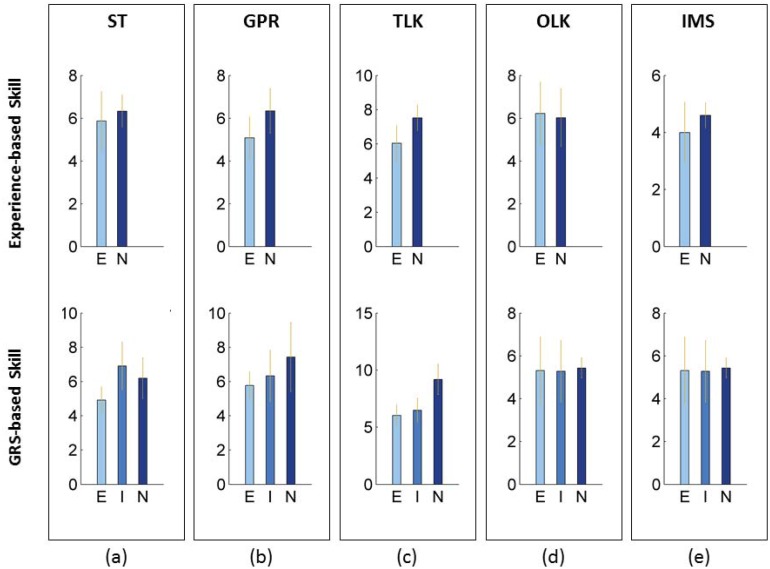
Total gesture counts within individual maneuvers by skill level. ST = suture throw; GPR = grasp-pull-run suture through tissue; OLK = one-loop knot; TLK = two-loop knot; IMS = inter-maneuver segment; E = expert; I = intermediate; N = novice. The error bars illustrate 95% bootstrap intervals. The Y-axis show the total number of gestures performed within each maneuver. The bar height represents the mean value, and the error bars represent 95% bootstrap intervals. We observed statistically significant differences in the total gesture counts for ST using a GRS-based skill definition, and for GPR and TLK using both experience- and GRS-based definitions for surgical skill.

Expert trials were executed with fewer gestures per task compared with trials in other skill categories. The difference in the total numbers of gestures between trials by expert surgeons (experience-based skill definition) and trials by novice surgeons was statistically significant (Fig 3, Table B in S1 File). Trials graded as expert by GRS were also performed using fewer gestures than trials graded as intermediate or novice. Differences in the total number of gestures were statistically significant only when comparing trials in the expert GRS category with those in the remaining two categories.

Trials by expert surgeons (experience-based skill definition) involved fewer error gestures than trials by novice surgeons; the difference between the groups was statistically significant. Similarly, trials graded as expert or intermediate by GRS were performed with fewer error gestures than trials by novice surgeons; the differences in numbers of error gestures between the novice GRS category and the remaining two categories were statistically significant (Fig 3, Table B in S1 File).

**Fig 4:**
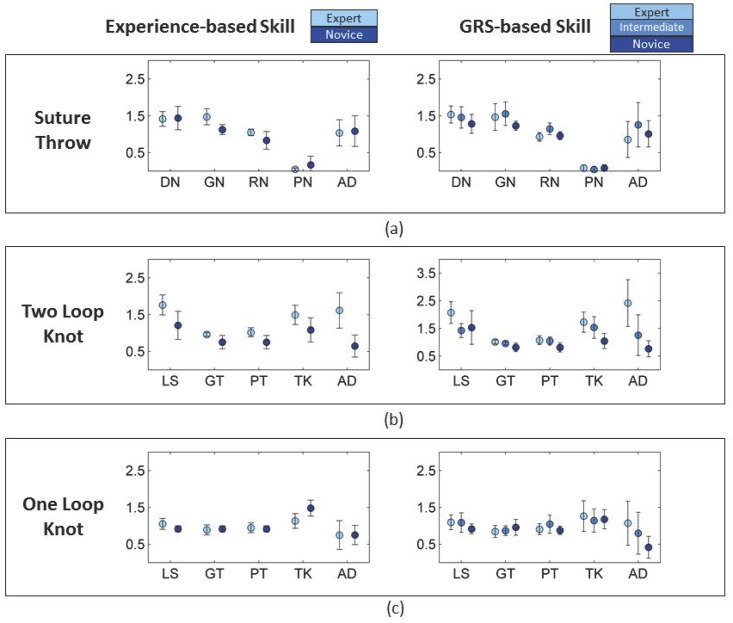
Counts for specific gestures within maneuvers by skill level. DN = drive needle; GN = grasp needle; RN = rotate needle out of tissue; PN = pull needle; AD = adjustment gestures; LS = loop suture; GT = grasp suture tail through loop; PT = pull suture tail through loop; TK = pull ends of suture to tighten knot. The Y-axis shows the counts of a particular gesture (X-axis) performed within each maneuver. The dots in the Fig 4 represent mean values and the bars represent 95% bootstrap intervals. We observed statistically significant differences in the counts for ST_DN and OLK_TK between expert and novice trials by GRS-based definition for skill, for ST_GN, ST_RN, TLK_LS, TLK_GT, TLK_PT, TLK_TK, and TLK_AD between expert and novice trials by both experience- and GRS-based definitions for skill.

Expert trials were executed with fewer (total number of) gestures within individual maneuvers comprising the task compared with trials in other skill categories. Per the experience-based skill definition, all but one maneuver (OLK) that we examined were performed using fewer gestures in expert trials compared with novice trials. The differences between expert and novice trials in the number of gestures within maneuvers were statistically significant for GPR and TLK. Per the GRS-based skill definition, all maneuvers in expert trials were executed with fewer gestures than novice or intermediate trials (Fig 4). The differences between trials in the expert and novice GRS categories in the number of gestures within maneuvers were statistically significant for ST, TLK, and IMS.

**Fig 5:**
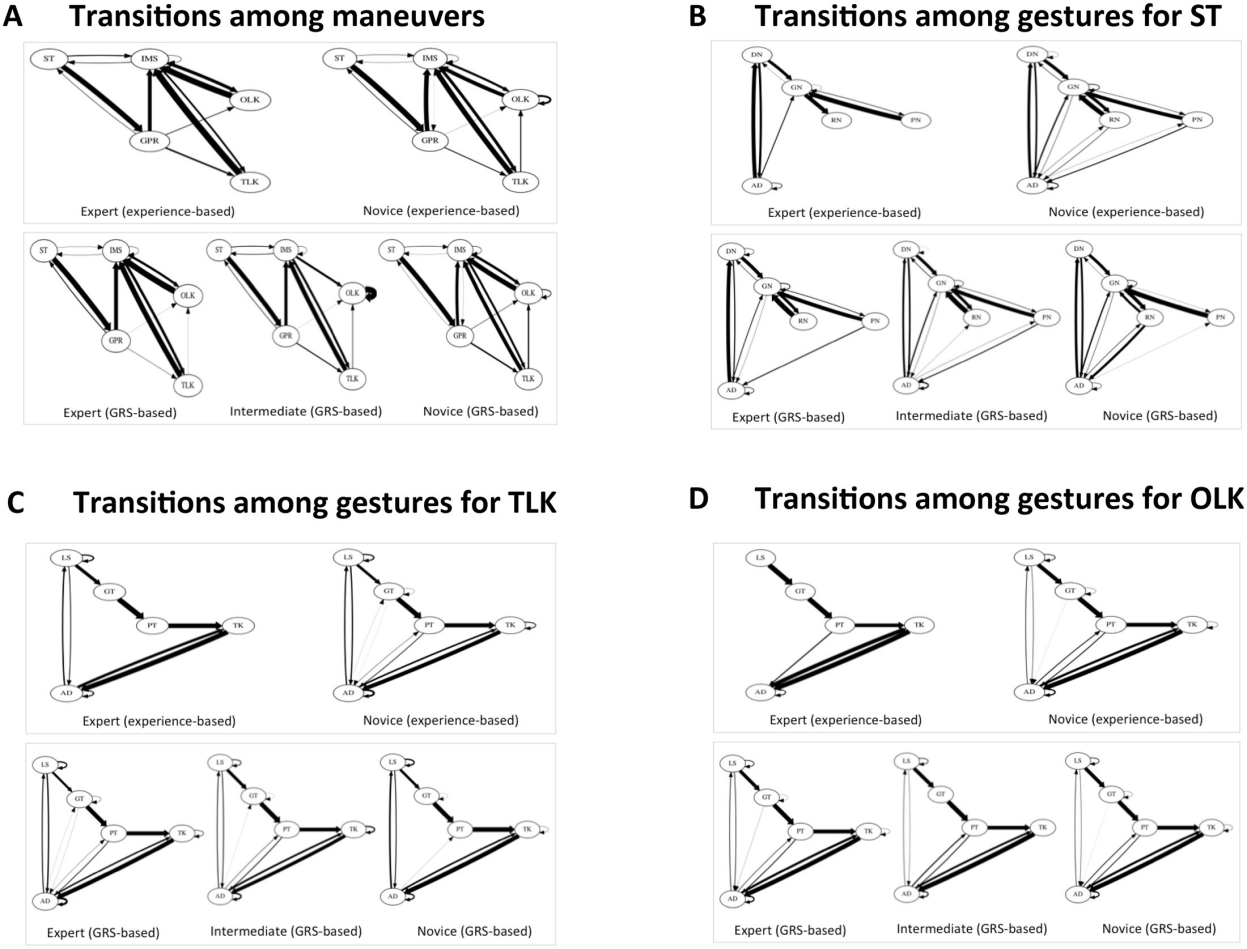
State flow plots showing transitions among maneuvers in a surgeon’s knot and among gestures in its constituent maneuvers by skill level. ST = suture throw; IMS = inter-maneuver segment; GPR = grasp-pull-run suture through tissue; OLK = one-loop knot; TLK = two-loop knot; GRS = global rating score; DN = drive needle; GN = grasp needle; RN = rotate needle out of tissue; PN = pull needle; AD = adjustment gestures; LS = loop suture; GT = grasp suture tail through loop; PT = pull suture tail through loop; TK = pull ends of suture to tighten knot. An arrow in the Fig 5 represents a transition from a maneuver shown at the tail to the maneuver shown at the head of the arrow. The thickness of the arrow corresponds to the fraction of total transitions arising out of the maneuver from which the arrow originates.

Certain gestures within maneuvers were performed less frequently in expert trials compared with novice trials. Using an experience-based skill definition, expert trials were executed with statistically significantly fewer GN and RN gestures in ST, and all gestures (LS, GT, PT, TK, and AD) in TLK. The differences in number of gestures within maneuvers were not statistically significant for most gestures comprising OLK. However, OLK in expert trials appeared to have been performed with more TK gestures than novice trials (Fig 5). Using a GRS-based skill definition, our findings for the numbers of gestures within maneuvers were consistent with those observed using an experience-based skill definition (Table C in S1 File).

State flow plots demonstrate differences in both occurrence and frequency of certain transitions by skill level. Experts performed the task using fewer transitions among maneuvers compared with novice surgeons. Some transitions were only observed in trials by novice surgeons, for example, a direct transition between an OLK and another OLK and a direct transition between a TLK and an OLK. Maneuver-level state flow plots illustrate that experts used fewer transitions between gestures to perform all three maneuvers we examined—ST, TLK, and OLK compared with novice surgeons (Fig 5). Both task-level and maneuver-level state flow plots of expert trials employed fewer AD compared with state flow plots of novice trials (Fig 5). Some transitions involving AD were unique to state flow plots of trials performed by novice surgeons (experience-based skill definition) for all three maneuvers.

Transitions among maneuvers and among gestures within maneuvers were quantitatively different between trials performed by expert and novice surgeons based on the Hellinger distance. This objective measure comparing expert and novice trials will equal zero when the task is executed using an identical sequence of activity segments. The Hellinger distance comparing expert and novice trials (experience-based skill definition) was 0.12 (95% BI = 0.00 to 0.38) for transitions emanating from ST, 0.29 (95% BI = 0.07 to 0.51) for transitions emanating from TLK, 0.43 (95% BI = 0.00 to 1.77) for transitions emanating from OLK, 0.19 (95% BI = 0.03 to 0.34) for transitions emanating from GPR, and 0.19 (95% BI = 0.00 to 0.46) for transitions emanating from IMS. The differences between expert and novice trials were more striking for transitions among gestures within individual maneuvers. The Hellinger distances for transitions emanating from all gestures within ST, TLK, and OLK were statistically significant, indicating that expert transitions between gestures within individual maneuvers were different from that of novices (Table D in S1 File).

Experts performed the task using a more predictable sequence of maneuvers compared with novice surgeons but the 95% BI included the null value suggesting that the difference was not statistically significant. The difference in the conditional entropy for probability distribution of transitions among maneuvers for trials by expert and novice surgeons was -0.24 (95% BI = -0.95 to 0.47).

## Discussion

We conducted a systematic descriptive analysis of the structure of activity for a simple surgical task to demonstrate differences in how expert surgeons perform the task compared with novice surgeons. Our reliability analysis of annotating surgical activity segments using a hierarchical task segment vocabulary indicates that independent annotation of at least a fraction of data is necessary to accurately establish reliability of different individuals using a given activity segment vocabulary.

Our findings on counts of activity segments in a surgical task and transitions among segments are consistent with subjective expert evaluation of surgical skill. Experts (assessed using both experience- and GRS-based definitions for skill) performed the task using fewer gestures and made fewer errors in gestures than novices. Trials in the intermediate skill category were performed with no more errors than expert trials but required a larger number of gestures than expert trials (Fig 3). This suggests that evaluation of operative performance and similarly, teaching and acquisition of technical skill, may be done at the gesture level.

In addition, objectively studying a surgical task at the gesture level was more discriminative of skill than at the maneuver level. Expert and novice trials were less distinct from each other when analyzing maneuvers in comparison to gestures based on the counts and visual examination of transitions. Maneuvers are large segments of activity comprising many gestures and thus variability in style and technique may have minimized differences in numbers of activity segments across skill categories. In contrast, gestures are atomic segments of activity and reflect how well the surgeon performed the task. Gesture-level examination is consistent with how surgical skill is typically assessed—evaluating both what actions a surgeon performs and how they perform them.

Gesture-based instruction may be critical for early stages of surgical skills acquisition. Typically, trainees learn to perform procedures by watching other surgeons in the operating room directly or through video footage, with or without additional expert instruction/commentary. Novice surgeons usually are able to observe and describe surgical tasks in terms of maneuvers. For example, for a suturing and knot-tying task, novice surgeons can easily articulate the constituent steps or maneuvers e.g., throw needle across the incision, run suture through the tissue, and tie a knot. However, it may be harder for them to both observe and articulate the actual gestures within each maneuver, e.g., drive needle through tissue, rotate needle out of tissue, make suture loop around needle-driver, as well as how these gestures are performed. This is consistent with our findings that although novice surgeons performed maneuvers in a similar manner to experts, the gestures within the maneuvers were clearly different in both counts and transitions. The relevance of gesture-based instruction is also mirrored in surgical teaching—when surgical technique is being taught by an expert surgeon, it is not merely the steps of the technique but also how the steps should be performed, i.e., how the tools or hands should move in a certain way for specific, atomic (gesture) segments of the task.

Our findings are limited in applicability as our study consisted of a small sample of surgeons. We also did not consider how well the individual gestures and maneuvers were performed. Further, we did not compare differences in surgical activity observed using our hierarchical, semantic vocabulary with what may have been observed using other approaches to decompose surgical activity, for example, using a vocabulary of event-based labels.

Segmenting surgical activity serves multiple purposes. Our results suggest that decomposing surgical activity either manually or using automated tools contains information on technical skill necessary to provide targeted feedback to trainees. For example, targeted feedback to trainees may focus on where in the task they performed more error gestures or they did not move as efficiently as expert surgeons. Skill acquisition in surgical trainees may be monitored through an objective evaluation of errors and efficiency of motion within and across activity segments (number of gestures, errors, and transitions). Segmentation may also be used to train automated algorithms to efficiently catalog a large amount of data from a long surgical procedure into its constituent parts for easy retrieval. Such a catalog can serve as an educational resource for surgical trainees.

The optimal granularity of surgical activity decomposition (e.g., maneuvers vs. gestures) that is useful for teaching purposes needs to be determined. A vocabulary to decompose surgical activity may be specified using either semantic or event-based labels [23–26]. A semantic vocabulary is specified based on expert knowledge of the surgical task [23–25]. Semantic labels describe meaningful movements performed by the surgeon’s hands or tools and are used for annotating surgical activity. Consequently, semantic labels may be useful for providing targeted feedback to trainees. For example, trainee feedback may refer to efficiency of motion while doing a two-loop knot and how to avoid specific error gestures they made while performing a maneuver. An event-based vocabulary is specified using data on changes in pose, position, or the state of the surgical tools, for example, whether a needle-driver is open or closed [26]. Event-based labels may not be useful for providing targeted feedback to surgical trainees because the labels are data-derived and represent abstract segments of activity.

Accurate and automated recognition of surgical activity segments is necessary for tools and technology that use the segmentation for objective technical skill assessment to be widely adopted. Current approaches to objective surgical technical skill assessment that use semantic decomposition of surgical activity depend upon manual annotation by watching videos of task performance [23,24,27]. Existing technology to automatically decompose surgical activity into its constituent semantic segments is moderately accurate [9,23–25,28–32]. Whether the utility of metrics, statistical models, or technological tools that use segmented surgical activity for objective skill assessment is affected by the accuracy of the automatic segmentation technology is yet to be studied.

In conclusion, our findings demonstrate that activity segments (maneuvers and gestures) differ by level of surgical skill both in counts and transitions. State flow plots of transitions between activity segments may be useful for providing targeted feedback to surgical trainees. Examining individual gestures in a task can be informative for evaluating surgical skill and for providing targeted feedback in surgical skills training.

## Supporting Information

S1 File Supplementary Tables. Inter-annotator reliability of manual annotation (Cohen’s kappa) using our hierarchical semantic vocabulary (**Table A**). Estimated total counts for maneuvers and gestures in the study task (**Table B**). Estimated counts for gestures within individual maneuvers comprising the study task (**Table C**). Hellinger distances for transitions emanating from gestures within maneuvers comprising the study task (**Table D**). Gestures in vocabulary corresponding to those listed in S1 Text (**Table E**). Maneuvers in vocabulary corresponding to those listed in S1 and S2 Text (**Table F**).
(DOCX)

S1 Text Gestures. Table with data on gestures; columns indicate trial number; gesture identifier; whether gesture was performed in error (no error = 0; error = 1); and corresponding row in the maneuver dataset for the gesture.
(TXT)

S2 Text Maneuvers. Table with data on maneuvers; columns indicate trial number; maneuver identifier; and whether maneuver was incomplete (complete = 0; incomplete = 1).
(TXT)

S3 Text Skill Assessments. Table with data on skill; columns indicate trial number; experience-based skill class (novice = 0; expert = 1); and total global rating score.
(TXT)
