# Supplementary material for: Analysis of the Structure of Surgical Activity for a Suturing and Knot-Tying Task
Source: PLoS One. 2016 Mar 7;11(3):e0149174. doi: 10.1371/journal.pone.0149174 (PMC4780814; doi:10.1371/journal.pone.0149174)
Supplement: S1 File — Inter-annotator reliability of manual annotation (Cohen’s kappa) using our hierarchical semantic vocabulary (Table A). Estimated total counts for maneuvers and gestures in the study task (Table B). Estimated counts for gestures within individual maneuvers comprising the study task (Table C). Hellinger distances for transitions emanating from gestures within maneuvers comprising the study task (Table D). Gestures in vocabulary corresponding to those listed in S1 Text (Table E). Maneuvers in vocabulary corresponding to those listed in S1 and S2 Text (Table F). (DOCX) [file pone.0149174.s001.docx]

**S1 File. Supplementary Tables**

**Table A**

**Title**: Inter-annotator reliability of manual annotation (Cohen’s kappa) using our hierarchical semantic vocabulary

| **Maneuvers** | | |
| --- | --- | --- |
|  | **Independent annotation**  **Mean (95% CI)** | **Verification of annotation**  **Mean (95% CI)** |
| All maneuvers | 0.99 (0.98 to 1.00) | Not performed |
| **Gestures** | | |
| Gestures executed with the first needle-driver | 0.79 (0.76 to 0.82) | 0.86 (0.83 to 0.89) |
| Gestures executed with the second needle-driver | 0.76 (0.73 to 0.79) | 0.84 (0.81 to 0.87) |
| Gestures executed with both needle-drivers | 0.75 (0.71 to 0.79) | 0.90 (0.88 to 0.92) |
| Camera movement and zoom | 1.00 (1.00 to 1.00) | 0.99 (0.98 to 1.00) |

**Legend**: CI = confidence interval

**Table B**

**Title**: Estimated total counts for maneuvers and gestures in the study task

|  |  | **Experience-based skill definition** | **GRS-based skill definition (#1)** | | |
| --- | --- | --- | --- | --- | --- |
|  | **Expert** | **Novice** | **Expert** | **Intermediate** | **Novice** |
| Maneuvers (total) | 6.46; 95% BI = (6.04 to 6.87) | 6.76; 95% BI = (6.32 to 7.19) | 6.02; 95% BI = (5.40 to 6.64) | 6.62; 95% BI = (5.97 to 7.27) | 6.93; 95% BI = (6.45 to 7.41) |
| Incomplete maneuvers | 0.17; 95% BI = (0.00 to 0.34) | 0.37; 95% BI = (0.26 to 0.48) | 0.29; 95% BI = (0.13 to 0.46) | 0.27; 95% BI = (0.08 to 0.45) | 0.33; 95% BI = (0.20 to 0.47) |
| Gestures (total) | 26.29; 95% BI = (25.21 to 27.38) | 31.30; 95% BI = (29.05 to 33.55) | 25.60; 95% BI = (24.87 to 26.34) | 30.29; 95% BI = (26.93 to 33.64) | 33.55; 95% BI = (29.24 to 37.85) |
| Error gestures | 1.00; 95% BI = (0.61 to 1.39) | 2.84; 95% BI = (2.30 to 3.37) | 1.73; 95% BI = (0.81 to 2.65) | 1.82; 95% BI = (1.08 to 2.56) | 3.43; 95% BI = (2.56 to 4.30) |
| ST (total) | 5.88; 95% BI = (4.50 to 7.25) | 6.33; 95% BI = (5.58 to 7.08) | 4.92; 95% BI = (4.14 to 5.69) | 6.91; 95% BI = (5.53 to 8.29) | 6.20; 95% BI = (5.01 to 7.38) |
| GPR (total) | 5.08; 95% BI = (4.10 to 6.07) | 6.34; 95% BI = (5.30 to 7.38) | 5.77; 95% BI = (4.97 to 6.57) | 6.33; 95% BI = (4.83 to 7.83) | 7.43; 95% BI = (5.42 to 9.43) |
| TLK (total) | 6.04; 95% BI = (4.98 to 7.10) | 7.52; 95% BI = (6.76 to 8.28) | 6.02; 95% BI = (5.08 to 6.97) | 6.48; 95% BI = (5.42 to 7.54) | 9.18; 95% BI = (7.85 to 10.50) |
| OLK (total) | 6.22; 95% BI = (4.76 to 7.69) | 6.02; 95% BI = (4.67 to 7.37) | 5.31; 95% BI = (3.72 to 6.90) | 5.28; 95% BI = (3.84 to 6.72) | 5.43; 95% BI = (4.97 to 5.89) |
| IMS (total) | 4.00; 95% BI = (2.94 to 5.06) | 4.60; 95% BI = (4.16 to 5.04) | 4.00; 95% BI = (3.21 to 4.79) | 4.18; 95% BI = (3.47 to 4.89) | 5.00; 95% BI = (4.31 to 5.69) |

**Legend**: BI = bootstrap interval; ST = Suture throw; GPR = Grasp-pull-run suture through tissue; TLK = Two-loop knot; OLK = One-loop knot; IMS = Inter-maneuver segment

**Table C**

**Title**: Estimated counts for gestures within individual maneuvers comprising the study task

|  | **Experience-based skill definition** | | **GRS-based skill definition (#1)** | | |
| --- | --- | --- | --- | --- | --- |
|  | **Expert** | **Novice** | **Expert** | **Intermediate** | **Novice** |
| ST_DN | 1.44; 95% BI = (1.12 to 1.76) | 1.41; 95% BI = (1.21 to 1.61) | 1.28; 95% BI = (1.02 to 1.54) | 1.46; 95% BI = (1.16 to 1.75) | 1.54; 95% BI = (1.30 to 1.77) |
| ST_GN | 1.12; 95% BI = (0.99 to 1.26) | 1.47; 95% BI = (1.26 to 1.69) | 1.23; 95% BI = (1.11 to 1.35) | 1.56; 95% BI = (1.24 to 1.87) | 1.46; 95% BI = (1.10 to 1.83) |
| ST_RN | 0.83; 95% BI = (0.59 to 1.07) | 1.05; 95% BI = (0.96 to 1.14) | 0.96; 95% BI = (0.85 to 1.07) | 1.14; 95% BI = (0.98 to 1.30) | 0.93; 95% BI = (0.81 to 1.05) |
| ST_PN | 0.17; 95% BI = (0.00 to 0.41) | 0.05; 95% BI = (0.01 to 0.09) | 0.08; 95% BI = (0.00 to 0.18) | 0.04; 95% BI = (0.00 to 0.12) | 0.08; 95% BI = (0.00 to 0.17) |
| ST_AD | 1.08; 95% BI = (0.67 to 1.50) | 1.04; 95% BI = (0.68 to 1.39) | 1.01; 95% BI = (0.66 to 1.37) | 1.26; 95% BI = (0.65 to 1.86) | 0.86; 95% BI = (0.37 to 1.34) |
| TLK_LS | 1.21; 95% BI = (0.83 to 1.59) | 1.76; 95% BI = (1.49 to 2.03) | 1.53; 95% BI = (0.92 to 2.14) | 1.42; 95% BI = (1.16 to 1.68) | 2.07; 95% BI = (1.68 to 2.46) |
| TLK_GT | 0.75; 95% BI = (0.57 to 0.93) | 0.96; 95% BI = (0.91 to 1.01) | 0.81; 95% BI = (0.66 to 0.97) | 0.96; 95% BI = (0.90 to 1.01) | 1.01; 95% BI = (0.94 to 1.09) |
| TLK_PT | 0.75; 95% BI = (0.57 to 0.93) | 1.02; 95% BI = (0.89 to 1.14) | 0.81; 95% BI = (0.65 to 0.98) | 1.04; 95% BI = (0.90 to 1.19) | 1.07; 95% BI = (0.91 to 1.23) |
| TLK_TK | 1.08; 95% BI = (0.75 to 1.41) | 1.49; 95% BI = (1.23 to 1.75) | 1.04; 95% BI = (0.77 to 1.31) | 1.53; 95% BI = (1.14 to 1.92) | 1.73; 95% BI = (1.36 to 2.09) |
| TLK_AD | 0.65; 95% BI = (0.35 to 0.94) | 1.61; 95% BI = (1.13 to 2.09) | 0.76; 95% BI = (0.47 to 1.05) | 1.26; 95% BI = (0.52 to 1.99) | 2.42; 95% BI = (1.57 to 3.26) |
| OLK_LS | 0.92; 95% BI = (0.84 to 1.00) | 1.05; 95% BI = (0.90 to 1.20) | 0.92; 95% BI = (0.78 to 1.05) | 1.09; 95% BI = (0.82 to 1.35) | 1.10; 95% BI = (0.89 to 1.30) |
| OLK_GT | 0.92; 95% BI = (0.84 to 1.00) | 0.89; 95% BI = (0.75 to 1.02) | 0.96; 95% BI = (0.74 to 1.17) | 0.87; 95% BI = (0.74 to 1.00) | 0.85; 95% BI = (0.68 to 1.01) |
| OLK_PT | 0.92; 95% BI = (0.84 to 1.00) | 0.95; 95% BI = (0.81 to 1.08) | 0.88; 95% BI = (0.76 to 0.99) | 1.04; 95% BI = (0.80 to 1.29) | 0.90; 95% BI = (0.75 to 1.06) |
| OLK_TK | 1.48; 95% BI = (1.26 to 1.70) | 1.13; 95% BI = (0.93 to 1.33) | 1.18; 95% BI = (0.91 to 1.44) | 1.14; 95% BI = (0.83 to 1.46) | 1.26; 95% BI = (0.85 to 1.68) |
| OLK_AD | 0.75; 95% BI = (0.49 to 1.01) | 0.75; 95% BI = (0.36 to 1.14) | 0.42; 95% BI = (0.12 to 0.71) | 0.80; 95% BI = (0.23 to 1.37) | 1.07; 95% BI = (0.47 to 1.67) |

**Legend**: BI = bootstrap interval; ST = Suture throw; GPR = Grasp-pull-run suture through tissue; TLK = Two-loop knot; OLK = One-loop knot; IMS = Inter-maneuver segment

**Table D**

**Title**: Hellinger distances for transitions emanating from gestures within maneuvers comprising the study task

| **Transitions emanating from:** | **Hellinger distance (95% bootstrap interval)** |
| --- | --- |
| Gestures within suture throw (ST) | |
| DN | 0.15 (0.06 to 0.24) |
| GN | 0.26 (0.08 to 0.44) |
| RN | 0.71 (0.71 to 0.71) |
| PN | 0.32 (0.32 to 0.32) |
| AD | 0.26 (0.07 to 0.44) |
| Gestures within two-loop knot (TLK) | |
| LS | 0.09 (0 to 0.18) |
| GT | 0.16 (0.04 to 0.27) |
| PT | 0.27 (0.19 to 0.35) |
| TK | 0.3 (0.18 to 0.43) |
| AD | 0.25 (0.14 to 0.36) |
| Gestures within one-loop knot (OLK) | |
| LS | 0.34 (0.26 to 0.41) |
| GT | 0.17 (0.09 to 0.26) |
| PT | 0.04 (0 to 0.28) |
| TK | 0.17 (0 to 0.39) |
| AD | 0.41 (0.27 to 0.55) |

**Legend**: DN = drive needle; GN = grasp needle; RN = rotate needle out of tissue; PN = pull needle out of tissue; AD = adjustment gestures; LS = loop suture; GT = grasp tail of suture through loop; PT = pull tail of suture through loop; TK = pull ends of suture taut

**Table E**

**Title**: Gestures listed in S1 Text

**First needle-driver (ND) gestures**

05 Align needle perpendicular to skin 1^st^ ND

06 Drive needle using 1^st^ ND to enter skin

08 Continue driving needle using 1^st^ ND through skin

11 Grab needle using 1^st^ ND

12 Pull needle/suture using 1^st^ ND

20 Grab suture tail using 1^st^ ND in knot tying

21 Pull suture tail using 1^st^ ND through knot

22 Grab suture 1^st^ ND

23 Rotate needle using 1^st^ ND through skin

26 Grab skin using 1^st^ ND

**Second ND gestures**

01 Reach needle with 2^nd^ ND

07 Grab skin using 2^nd^ ND

09 Rotate needle using 2^nd^ ND through skin

10 Grab needle using 2^nd^ ND

13 Grab suture using 2^nd^ ND

16 Grab suture tail using 2^nd^ ND in knot tying

17 Pull suture tail using 2^nd^ ND through knot

28 Pull needle/suture using 2^nd^ ND

**Both ND gestures**

02 Transfer needle using 2^nd^ ND to 1^st^ ND

03 Align needle on 1^st^ ND

14 Transfer suture using 2^nd^ ND to 1^st^ ND

15 Rotate suture twice using 1^st^ ND around 2^nd^ ND

18 Pull ends of suture taut

19 Rotate suture once using 2^nd^ ND around 1^st^ ND

25 Rotate suture once using 1^st^ ND around 2^nd^ ND

27 Rotate suture twice using 2^nd^ ND around 1^st^ ND

29 Transfer suture using 1^st^ ND to 2^nd^ ND

30 Transfer needle using 1^st^ ND to 2^nd^ ND

**Camera gestures**

04 Camera zoom in or out

24 Camera rotation

**Table F**

**Title**: Maneuvers listed in S1 and S2 Text

01 Suture throw

02 Knot using 1^st^ needle-driver (ND) with two loops

03 Knot using 1^st^ ND with one loop

04 Knot using 2^nd^ ND with two loops

05 Knot using 2^nd^ ND with one loop

06 Inter-maneuver segment

07 Pull/run suture out of tissue
